# Minimally invasive approach to Herlyn-Werner-Wunderlich syndrome with giant endometriotic cyst: a case report

**DOI:** 10.11604/pamj.2023.44.79.32667

**Published:** 2023-02-08

**Authors:** Saket Kumar, Madhukar Dayal, Abhay Kumar, Nidhi Singh, Vijay Prakash Singh

**Affiliations:** 1 Department of Surgical Gastroenterology, Indira Gandhi Institute of Medical Sciences, Patna, Bihar, India,; 2 Department of Radiology, BIG Apollo Spectra Hospitals, Agamkuan, Patna, India,; 3 Department of General, Gastrointestinal and Laparoscopic Surgery, BIG Apollo Spectra Hospitals, Agamkuan, Patna, India,; 4 Department of Anesthesiology, BIG Apollo Spectra Hospitals, Agamkuan, Patna, India,; 5 Department of Gastroenterology and Hepatology, BIG Apollo Spectra Hospitals, Agamkuan, Patna, India

**Keywords:** Renal agenesis, dysmenorrhea, Herlyn-Werner-Wunderlich syndrome, case report

## Abstract

Herlyn-Werner-Wunderlich syndrome is a rare congenital anomaly of the Mullerian and Wolffian ductal system, manifesting as a triad of dipelphys uterus, obstructed hemivagina, and ipsilateral renal agenesis. Patients usually remain asymptomatic till menarche and experience progressive dysmenorrhea, suprapubic lump, and/or features of infection (pyometra, pelvic collection, etc.) afterward. We hereby present a case of a young lady with Herlyn-Werner-Wunderlich syndrome with a large endometriotic cyst, likely arising from the right hemiuterus. She presented with dysmenorrhea and progressive abdominal distention for seven years. She was treated by laparoscopic ovarian cyst excision and right hemihysterectomy that relieved her symptoms.

## Introduction

Herlyn-Werner-Wunderlich syndrome (HWWS) is a rare congenital anomaly characterized by the triad of uterus didelphys, obstructed hemivagina, and ipsilateral renal agenesis. It is also known as Obstructed Hemivagina with Ipsilateral Renal Agenesis (OHVIRA) syndrome and results due to anomalous development of Müllerian and Wolffian ductal systems [1]. The exact incidence of HWWS is unknown, however, the studies have reported the incidence between 0.1% and 3.8% in the general population [2].

Symptoms usually develop after menarche and patients usually present with dysmenorrhea, pelvic pain, lower abdominal mass, and sometimes with intermenstrual bleeding secondary to haematocolpos and/or haematometra. Because it is rare, the diagnosis is often delayed, which increases the risk of complications such as endometriosis, infections, and secondary infertility [3,4]. We report here, a case of HWWS in an Indian lady with a giant right ovarian endometriotic cyst. The ovarian cyst and obstructed hemiuterus were treated laparoscopically.

## Patient and observation

**Patient information:** a 19-year-old lady presented with progressively increasing abdominal lump and dysmenorrhea for seven years. She attained menarche at the age of 12 years and her menstrual cycle was regular (28-30 days) with menstrual phase of four to five days. There was no history of fever, weight loss or anorexia. The past medical and surgical history was not significant.

**Clinical findings:** on examination, there was a large, non-tender abdominal lump arising from the pelvis and reaching up to the epigastrium. The patient was conscious, and oriented and her vital parameters were normal.

**Diagnostic assessment:** she was evaluated elsewhere where an abdominal ultrasound scan was done. The scan had revealed right renal agenesis with giant right ovarian cyst (13 x 21 x 23 cm) and bicornuate uterus. The right horn of the uterus was distended with hypodense fluid suggestive of haematometra. The ovarian cyst was reported to be of suspicious nature and a cross-sectional imaging test was advised for better characterization of the lesion. The left horn of the uterus was normal. The left kidney was normal in size and position. Intravenous urogram was done that revealed no contrast excretion from the right kidney. The left-sided kidney and ureter were normal.

**Figure 1:**
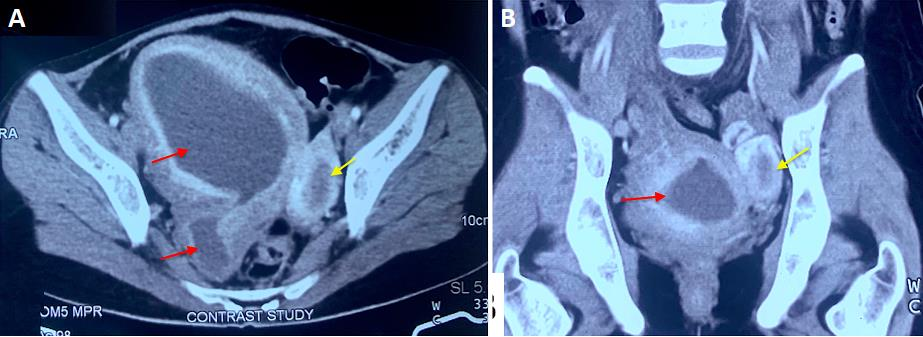
A) axial view CT scan image showing uterus didylphus with hematometra and hematocolpos of the right side (red arrows); the left horn was apparently normal (yellow arrow); B) coronal view

**Figure 2:**
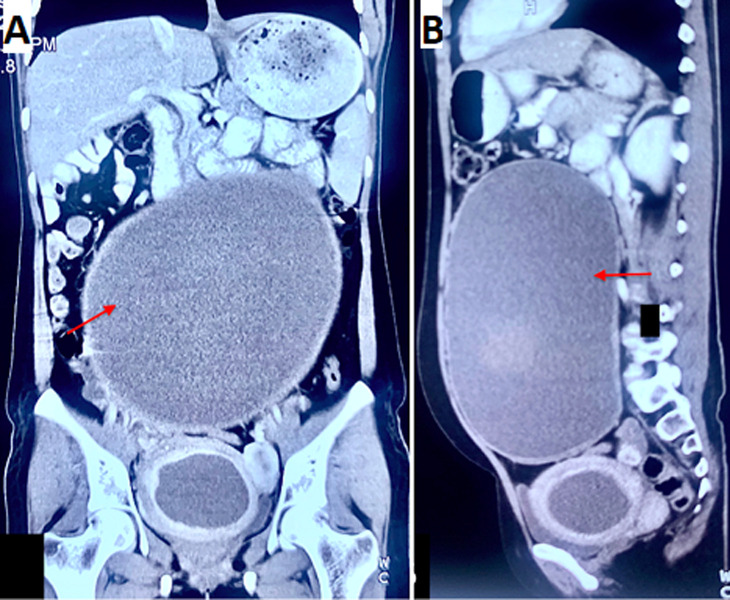
A) coronal view CT scan showing a large endometriotic cyst arising from the right ovary and reaching up to the epigastrium (red arrow); cyst was displacing the small bowel loops cranially; B) sagittal view

**Figure 3:**
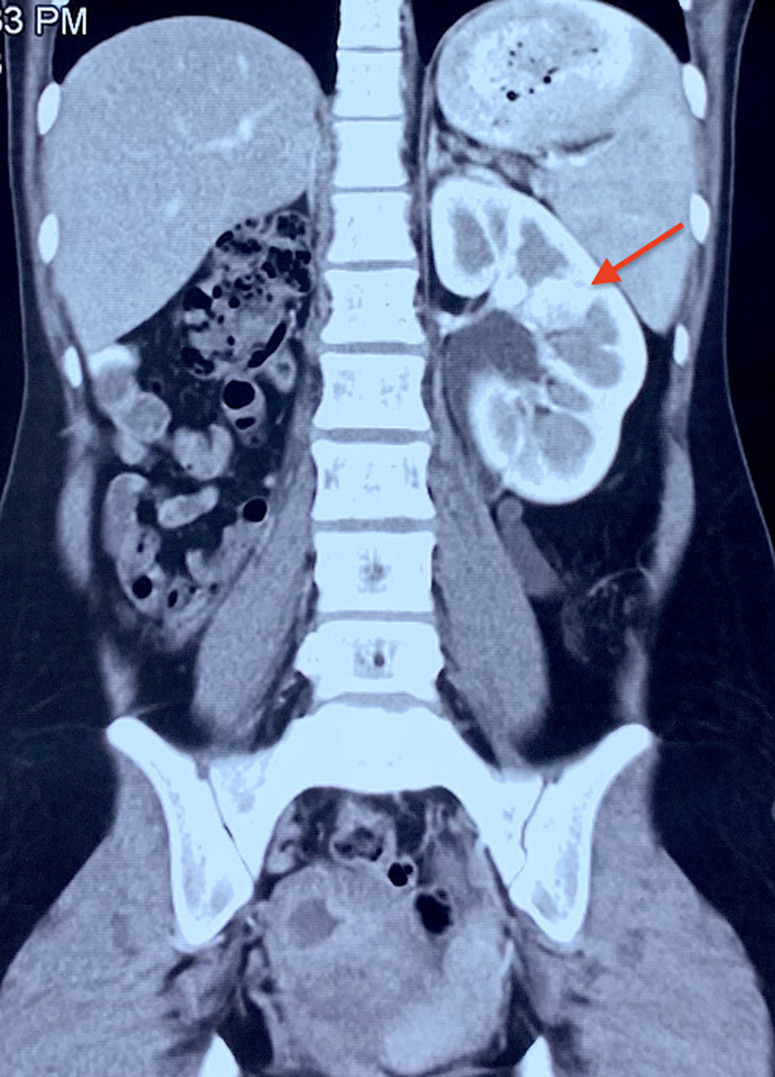
the right renal agenesis was noted on the computed tomography image; the left kidney was hypertrophic (arrow)

At our center, a contrast-enhanced computed tomogram scan of the abdomen was obtained that revealed uterine didelphys with distended right endometrial cavity along with obstructed right hemivagina and hematocolpos (Figure 1). A large ovarian cystic lesion (13 x 21 x 22 cm) was seen, extending up to the level of the pancreas, displacing the bowel loops (Figure 2). The cyst had no enhancing solid component indicative of malignancy and was likely benign. The right kidney could not be visualized (Figure 3). The routine blood investigations and tumor markers (beta-HCG, lactate dehydrogenase, alpha-fetoprotein, and CA-125) levels were within normal limits.

**Diagnosis:** based on the imaging findings of uterine didelphys, obstructed uterine horn and ipsilateral renal agenesis, a diagnosis of Herlyn-Werner-Wunderlich syndrome was confirmed. The patient was planned for laparoscopic ovarian cyst excision along with excision of obstructed right horn of the uterus.

**Figure 4:**
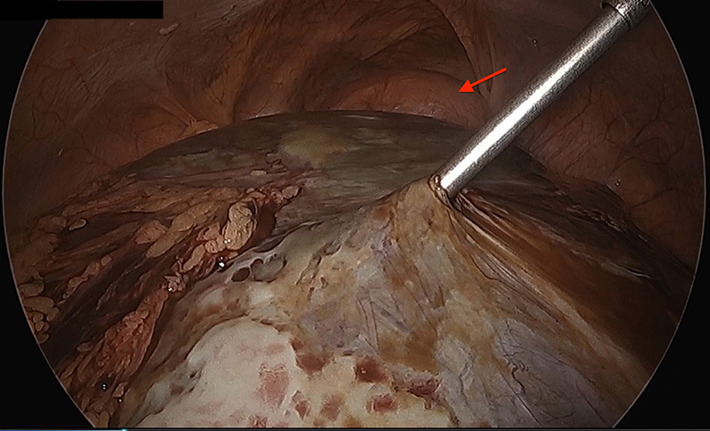
a large cyst arising from the pelvis was encountered during laparoscopy; cyst was aspirated completely; the distended right uterine horn then could be visualized in the pelvis (arrow)

**Figure 5:**
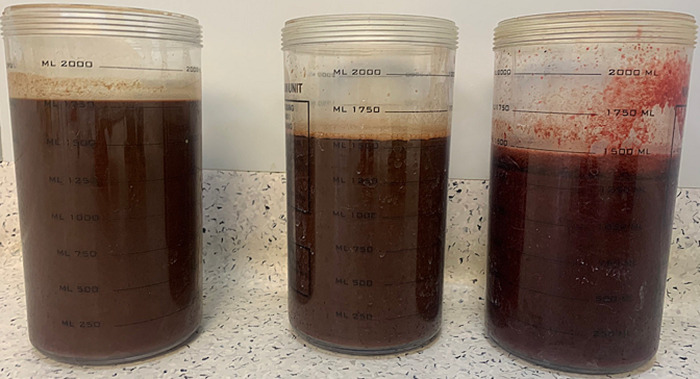
approximately 4 liters of chocolate-colored fluid was aspirated from the endometriotic cyst

**Figure 6:**
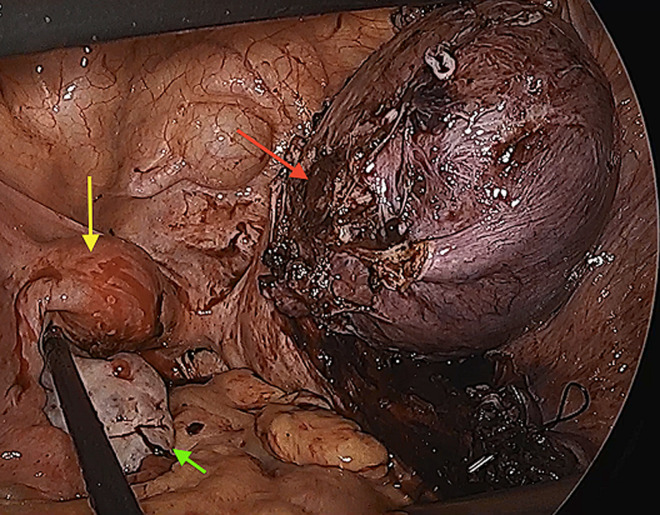
laparoscopic view of pelvis after removal of endometriotic cyst: uterus didelphys with distended right uterine horn (red arrow) and normal left horn (left horn); left ovary appeared normal (green arrow)

**Therapeutic interventions:** surgery was performed under general anesthesia with the patient in the supine position. Diagnostic laparoscopy revealed a large cystic lesion occupying most of the abdominal cavity (Figure 4). Another 5 mm port was placed in the right subcostal region. The cyst was then punctured in a controlled fashion and the content was aspirated; approximately four liters of chocolate-colored fluid was suctioned (Figure 5). The pelvis could be visualized after cyst decompression. Another 5 mm working port was placed in the right iliac fossa. The small bowel loops were moved out of the pelvic cavity, and the uterus and ovary were inspected. The cyst was found to be adherent to the right ovary. The bifid uterus was seen with a distended, enlarged right horn and a small left horn (Figure 6).

**Figure 7:**
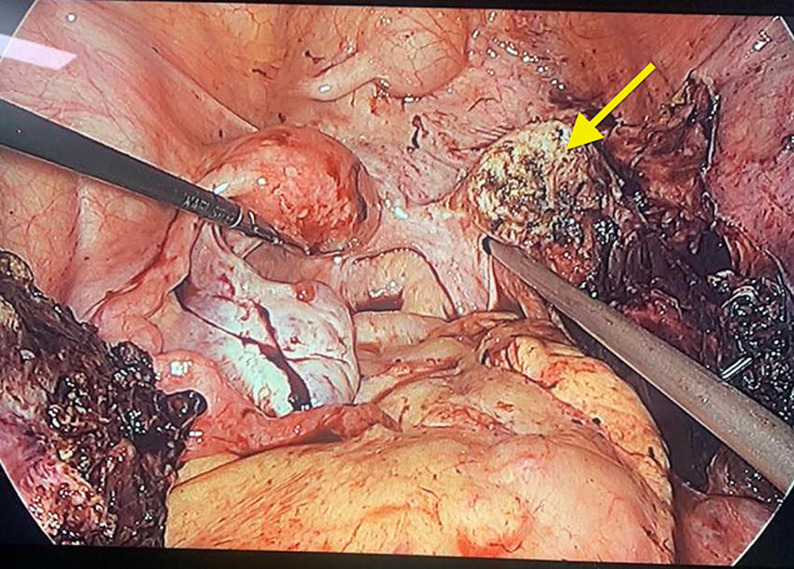
right hemihysterectomy was performed preserving the left uterine horn

The cyst was dissected off the ovary using ultrasonic shear. The distended right uterine horn was punctured using an aspiration needle and approximately 200 ml of altered blood was suctioned out. The obstructed right horn was then excised using an ultrasonic shear device and the stump was closed with polypropylene sutures (Figure 7). The cyst wall and right uterine horn were removed by extending one of the right iliac fossa port sites.

**Follow-up and outcome of interventions:** the patient had an uneventful postoperative recovery. She was discharged from the hospital on day three of surgery. On histopathological examination, the ovarian cyst was reported to be a benign endometriotic cyst and the excised uterine horn was reported to have adenomyosis. The patient remains symptom-free in the follow-up. She continues to have a normal menstrual cycle after the surgery.

**Informed consent:** written informed consent was obtained from the patient for the publication of this case report, accompanying images, and de-identified information.

## Discussion

Herlyn-Werner-Wunderlich syndrome (HWWS) is a rare congenital anomaly of the urogenital tract occurring due to anomalous development of Mullerian and Wolffian ducts. This syndrome is characterized by a constellation of anomalies namely uterine didylphys, obstructed hemivagina, and ipsilateral renal agenesis [1,2]. Herlyn-Werner described the association of renal agenesis with ipsilateral obstructed hemivagina in 1971, whereas Wunderlich described the triad of bicornuate uterus, obstructed hemivagina, and ipsilateral renal agenesis in 1976 [5].

During normal female embryogenesis, the paramesonephric ducts fuse in the midline giving rise to the uterus, cervix, and upper part of the vagina. In the HWWS the paramesonephric ducts fail to fuse together resulting in didylphic uterus. The non-fusion of the caudal part of the paired Mullerian duct and vaginal plate formed by the urogenital sinus produces a persistent complete or partial vaginal septum that results in obstructed hemivagina. The development of Mullerian ducts and Wolffian ducts is closely associated in the embryonic period. The prolonged obstruction of hemivagina in the developmental phase is postulated to result in ipsilateral renal agenesis. Renal agenesis occurs in approximately 40% of females with Müllerian duct anomalies [6]. The right side is affected twice more commonly as compared to the left side [7]. Our patient also had renal agenesis and hematometra of the right side. The exact etiopathogenesis and embryological basis of HWW syndrome remain obscure and are topics of further research.

Due to the lack of symptoms and normal-appearing external genitalia, the syndrome remains undiagnosed in early childhood. The patients typically present shortly after menarche with cyclical pelvic pain, secondary to hematocolpos. The pain may be of acute onset, severe type or chronic, low-grade pelvic and lower back type. Patients with HWWS generally have regular menstruation from the non-obstructed horn. However, on the obstructed side, retrograde drainage can happen, leading to dysmenorrhea. A long-term retrograde menstrual flow may result in secondary endometriosis, pelvic adhesions, and infections [6,8]. A progressive enlarging pelvic mass has also been described in the literature. In a large retrospective study by Tong *et al*. pelvic endometriosis was reported in approximately a fifth of all patients with HWWS. The ovarian endometrial cysts occurred ipsilateral to the vaginal septum in all the cases [9].

Our patient also had long-standing symptoms of dysmenorrhea and growing abdominal mass. She had developed a giant endometrial ovarian cyst, likely due to prolonged retrograde menstrual flow. The cyst contained four liters of chocolate-colored fluid. HWWS is usually diagnosed on ultrasonic examination of abdomen in symptomatic patients. The abnormal uterine morphology, ipsilateral renal agenesis, and hematometra or hematocolpos are easily picked up on ultrasound. In our patient, the ultrasound revealed all these findings along with a giant cyst arising from the pelvis. The cyst content was of mixed echogenicity and neoplastic etiology could not be ruled out. Hence, a contrast-enhanced computed tomography (CT) scan of the abdomen was obtained. The anomalies related to the uterus and right renal agenesis were confirmed. A large chocolate cyst in relation to the right ovary was seen. The cyst has no irregular or enhancing solid component indicative of malignancy. The diagnosis of HWWS was confirmed on the basis of characteristic findings, although the exact etiology of the ovarian cyst was not confirmed on imaging [10,11]. The classification of HWWS has been done into two types based on the vaginal morphology (Table 1) [12]. Our patient had type 1.1 HWWS.

**Table 1:** classification of Herlyn-Werner-Wunderlich syndrome based on morphology by Zhu *et al*.

Classes	Description
**Class 1**	**Complete obstruction of hemivagina**
	With blind hemivagina
	Cervico-vaginal atresia
**Class 2**	**Incomplete obstruction of hemivagina**
	Incompletely obstructed hemivagina
	With communicating uteri

Surgery is the most effective treatment to relieve the symptoms and retain the fertility of patients [6,7]. The treatment of HWWS includes resection of the obstructing vaginal septum or excision of the obstructed hemiuterus. It could be argued that surgical excision might be better as there have been reported cases of malignancy occurring in the ectopic uterus. Also, with excision, the possibility of recurrent fluid accumulation in the obstructed hemivagina after ipsilateral hemihysterectomy is averted [6,13]. The resection of the vaginal septum can also be performed by transabdominal (open or laparoscopic approach) or trans-hymenal approach. The patients can have normal coitus with a good chance of getting pregnant, although the incidence of abortion and premature delivery is high [14]. Our patient had symptoms for seven years and had an associated large ovarian cyst. She was planned for laparoscopic excision of the cyst and obstructed unilateral hysterectomy. The left uterus was properly preserved.

## Conclusion

Herlyn-Werner-Wunderlich syndrome is a rare anomaly that should be suspected in young females with unilateral renal agenesis or those gradually increasing dysmenorrhea and pelvic lump. Early diagnosis is paramount in avoiding complications such as hematometra, endometriosis, infections, and pelvic adhesions that may lead to secondary infertility. Diagnosis of HWWS is often delayed due to its rarity and non-specific presenting symptoms. In this context, a high degree of suspicion should be maintained for early diagnosis and timely management.
